# A Search for Optical Molasses in a Vapor Cell: General Analysis and Experimental Attempt

**DOI:** 10.6028/jres.094.038

**Published:** 1989

**Authors:** A. L. Migdall

**Affiliations:** National Institute of Standards and Technology, Gaithersburg, MD 20899

**Keywords:** laser cooling, optical molasses, vapor cell

## Abstract

We analyze the application of optical molasses to a thermal vapor cell to make and collect cold atoms. Such an arrangement would simplify the production of cold atoms by eliminating the difficulty of first having to produce and slow an atomic beam. We present the results of our calculations, computer models, and experimental work. As a guide for future work, general results are given to illustrate which fundamental parameters are most important in the production of cold atoms in a vapor cell.

## 1. Introduction

We have proposed using the technique of laser cooling to directly cool and collect gas atoms in a thermal vapor cell. This allows a high density region of very cold atoms to be built up [1]. This eliminates the difficulty of first having to produce and slow an atomic beam, and thus holds the promise of greatly simplifying the production of cold atoms. We present here an analysis of the processes involved in this proposal, as a guide for future work, and give the results of our attempts to realize it experimentally.

The proposal is to set up a region of optical molasses directly in an atomic vapor cell, allowing the accretion of a high density of cold atoms. The molasses consists of three pairs of counterpropagating laser beams tuned below resonance in an atomic vapor cell. This arrangement [2] of opposing light beams provides a strong damping or viscous force for slow atoms. This effect, which was first demonstrated with a cooled atomic beam several years ago [3], is well described in the literature [4,5] and so will only be discussed briefly here. Atoms that enter the molasses region with slow enough velocities have a high probability of being slowed and viscously captured before exiting the other side. The motion of these captured atoms becomes diffusive, because any velocity the atoms acquire in a particular direction (due to random scattering of photons) is quickly damped by the viscous force [3]. Because of this random walk type motion, these atoms remain in the molasses region for much longer times than they would have under ballistic motion at their original thermal velocities or even their ultimate cooled velocity (hence the term optical molasses). After a time, all the atoms in the cell with “catchable” velocities (most probably those with velocities less than some critical value) become mired in the molasses region. Thus the density of cold atoms in the molasses region builds up, as the lowest velocity atoms in the rest of the cell are depleted. The density of cold atoms continues to increase as the low velocity tail of the thermal distribution is replenished by processes that move the velocity distribution back toward thermal equilibrium. It is the fluorescence of the accumulated cold atoms that would be observed.

## 2. Theoretical Analysis

We calculate how fast the cold atom density should increase with time and estimate the processes limiting residence time in classical two-state molasses [5]. This is done with the help of computer models of the cooling process for the specific case of a sodium atom in a vapor cell containing a molasses region. In addition, we calculate the visibility of the effect by comparing the fluorescence signal expected from the cold atoms to the background signal that is also present. We also examine the general dependences of the signal size on the atomic parameters, so that comparisons can be made of the relative merits of trying to cool other atoms in a vapor cell or using other transitions.

The initial rate of increase of the cold atom density is governed by the rate at which capturable atoms enter the molasses region. The rate of entry (and subsequent capture) of atoms is just the flux Φ,

$$\Phi =\frac{1}{4}n_{c}V_{\text{cavg}}$$ (1)

integrated over the surface area of the molasses volume, where *n*_c_ is the density of catchable atoms and *V*_cavg_ is the average velocity of those atoms. The probability of capture versus velocity for a two-state atom entering the molasses region was determined by a Monte Carlo simulation. The use of this simple two-state model is justified in this situation, because during most of the capture process, the velocities correspond to detunings that are large relative to atomic linewidth. (It is important to note that it is the capture rate that is more important in estimating the final signal rather than the ultimate temperature of the molasses, since the residence time in the molasses is limited by other processes.) A 1-cm region of 3-D molasses is modeled, where each beam has a saturation parameter of one (defined as *I/I*_0_, where/is the light intensity and *I*_0_ is the on-resonance intensity which power broadens the natural transition width by a factor of $\sqrt{2}$). The laser frequency is tuned Γ_n_*/*2 below resonance, where Γ_n_ is the natural width of the cooling transition. For this model, the result shows that the catchable atoms are simply those atoms with velocities less than some maximum cutoff value, *V*_c_. In this case, the catchable density, *n*_c_ found by integrating the Maxwell-Boltzmann distribution from *V* = 0 to *V*_c_ (for *V*_c_< <√(*k*_B_T/M), where *k*_B_ is the Boltzmann constant, *T* is the gas temperature, and *M* is the atomic mass) is given by:

$$n_{c}=\frac{4}{3\sqrt{\pi }}n_{0}{\left(\frac{M}{2k_{B}T}\right)}^{3/2}V_{c}^{3},$$ (2)

where *n*_0_ is the total density of the thermal atoms in the cell. The average velocity of these atoms, found by performing a similar integration, is just equal to (3/4) *V*_c_. The fractional increase in the total density with time, *F*(*t*) (for *t* much less than the average time for leaving the molasses) equals the rate of new atoms getting caught in the region times *t* divided by the volume of a sphere of radius *R* and the original density, *n*_0_:

$$F\left(t\right)=\frac{\frac{1}{4}n_{c}\;V_{\text{cavg}}\;4\pi R^{2}}{n_{0}\frac{4}{3}\pi R^{3}}t=\frac{9\;n_{c}\;V_{c}}{16n_{0}\;R}t.$$ (3)

**Figure 1:**
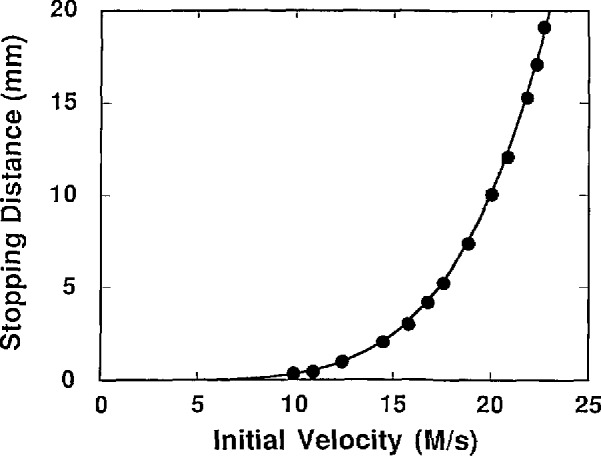
The distance required to bring a Na atom from its initial velocity to the point where its motion is completely diffusive. The points are the result of a model of 3-D molasses with each beam having a saturation parameter of one. The size of the molasses region needed to hold an atom for an extended time must be somewhat larger than the stopping distance, because the atom must stop away from the boundary of the region to remain caught for an appreciable time. The line is a fit to a power law, *aV*^b^. The best fit parameters are *a* = 4.7 × l0^−6^ and *b* = 4.87, very nearly the expected 5th power form.

To estimate this increase, we use the results of our computer simulations, including those shown in figure 1. This figure shows the general result that the average distance needed to snag an Na atom in uniform molasses is proportional to the 5th power of the initial velocity. (Note that the size of the molasses region required to catch and hold an atom would be somewhat larger than these stopping distances, since the atom must not stop right on the boundary if it is to remain caught for an appreciable length of time.) This velocity dependence agrees with the calculated dependence obtained by expanding to first order, the velocity dependence of the damping force for a red detuning of Γ_n_/2 and *kV*>Γ_n_, where *k* is the wave vector in wavenumbers of the laser. By integrating, we get the stopping distance, *D* as a function of velocity. The result of this calculation has the form

$$D≃\frac{Mk^{2}V_{c}^{5}}{5\hbar \Gamma_{n}^{4}}.$$ (4)

According to our simulations for Na atoms on typical trajectories traversing a molasses region consisting of the intersection of three 1-cm diameter beams, 20 m/s is roughly the critical velocity, below which the probability of stopping before exiting the other side is nearly unity. The fraction of atoms with velocities less than 20 m/s, as given by eq (2), is 5 × 10^−5^ of the total Na density at 75 °C.

**Figure 2:**
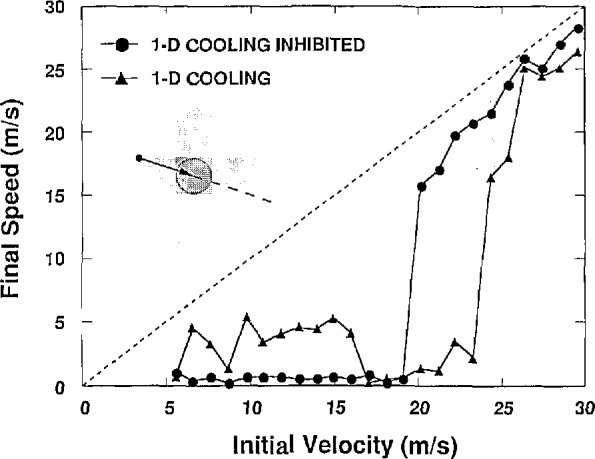
The final speed of Na atoms incident on the molasses region vs starting velocity. The molasses region has a central volume of 3-D damping surrounded by 1-D damping due to a single pair of beams. The insert shows the particular trajectory used, which passes through the 1-D region before entering the 3-D region. The circles are for the configuration with both laser frequencies in all beams. The triangles indicate that one laser frequency is in one pair of beams and the second frequency in the other two pairs. For this curve, only atoms with velocities in the range between 17 and 19 m/s were caught. Atoms in the range from 20 to 23 m/s were greatly slowed but did not in fact stop in the 3-D molasses region. The difference between the dashed line and the data points is the total slowing experienced by the atom.

We also investigated how the capture rate is affected by having 1-D molasses surrounding the central 3-D molasses region [6]. This is important because, before an atom enters the central region it is likely to pass through a region of only one pair of counterpropagating beams. In this region, only one component of velocity is damped, but it is just the component of velocity needed to reach the central region. Thus some atoms headed for the central region may be, in effect, deflected away, thereby reducing the velocity capture range. This process is modeled and found to produce a minimum velocity below which atoms on certain trajectories cannot reach the central volume. The circles in figure 2 show an example of a trajectory where only those atoms with velocities between 17 and 19 m/s are stopped in the molasses region. The trianglar points in figure 2 represent a nearly equivalent case to that of no surrounding 1-D molasses for reasons to be described later. 1-D molasses surrounding the central 3-D region should also produce a slight increase in the maximum catchable velocity, due to the extra length of the cooling region. Some effect of this is seen on those velocities just slightly too fast to be caught. The circular points show significant cooling of velocities in the 20 to 23 m/s range, whereas the triangular points show minimal cooling in that range. The number of atoms within this band of capturable velocities is about one-third of the number that is caught in the central 3-D molasses without the surrounding 1-D molasses. We describe a means to reduce this effect later.

What ultimately limits the maximum density that can be built up are the processes that remove cold atoms from the molasses. Atoms leave the region by diffusion, by aquiring a drift velocity due to imbalance of opposing beams [5], and by collisions with the thermal gas atoms which transfer a relatively large amount of kinetic energy. Here we estimate the time for each of these processes, assuming classical two-state optical molasses damping.

The time for an atom to random walk out of the molasses is proportional to *R*^2^, where *R* is a characteristic radius of the molasses region. Assuming for Na a random walk with a time step size equal to the velocity correlation time of 4 × l0^−5^ s and a velocity equal to the simple Doppler cooling limit of 50 cm/s, the average time to leave is long, about 0.45 s for a 1-cm diameter region. The calculated time [5] for an atom to drift out due to a beam intensity imbalance is also long, 0.25 s for a 1% intensity imbalance, *I/I*_0_ = 1, and a detuning of Γ_n_/2. Our final estimate is not hurt by using such a simple model of molasses since it is known to underestimate these times which, in our case, are already long enough not be the process limiting the final density buildup.

The time for a cold atom to be knocked out of the molasses depends on the density of the thermal gas atoms and their collision cross sections. The dominant cross section for this is the elastic cross section for collisions between ground-state and excited-state atoms. We estimate this cross section to be on the order of 10^−13^ to 10^−12^ cm^2^, which for Na at 75 °C, gives a time to be knocked out of the molasses of 1.0 to 0.1 s, assuming that half the atoms are in the excited state.

The visibility of the cold atom signal depends on the size of that signal relative to any background signal. A background signal exists because each of the laser beams is in resonance with a fraction of the uncooled atoms. This fraction is approximately equal to Γ_n_/Γ_D_ where Γ_D_ is the Doppler width of the resonance transition at the cell temperature. This will produce a sizable background fluorescence signal. The total background fluorescence signal will be about three to six times the signal due to a single beam, since the three pairs of beams are orthogonal, and the tuning is such that the six beams interact with nearly distinct velocity classes.

## 3. Signal Estimate

To understand better how cell molasses works, it is useful to estimate the signal size in terms of fundamental atomic parameters so that the estimate can easily be extended to an arbitrary atom. In this way, we can see best how to maximize the cold atom signal. To do this, we eliminate *V*_c_ and *n*_c_ from eq (3) by writing them in terms of the mass and transition width of the atom. Substituting eqs (2) and (4) into eq (3) and using *D* =2*R* we get

$$F\left(t\right)=3\;{\left(\frac{M}{2k_{B}T}\right)}^{3/2}\frac{{\left[\frac{10\hbar \Gamma_{n}^{4}}{Mk^{2}}\right]}^{4/5}}{4\sqrt{\pi }R^{1/5}}t$$ (5)

To determine the size of the signal expected from the increase in density of the cold atoms, we take the ratio of this density to the density of the hot atoms that are also fluorescing. The total fraction of hot atoms that are on resonance with any of the laser beams is estimated to be ~ 5Γ_n_/Γ_D_, for a laser tuned Γ_n_/2 below resonance. Since Γ_D_ is proportional to the thermal velocity of the atoms, the ratio S of the cold atom signal to the background fluorescence can be written as:

$$S\left(t\right)=\frac{F\left(t\right)}{5\Gamma_{n}/\left[2k{\left(\frac{2k_{B}T}{M}\ln2\right)}^{1/2}\right]}.$$ (6)

Substituting eq (5) into eq (6) and extracting just the proportionalities with respect to the atomic parameters and cell conditions, we find

$$S\left(t\right)\;\alpha \;M^{0.2}\;\Gamma_{n}^{2.2}\;R^{-0.2}\;T^{-1}.$$ (7)

From this form we can see that *S*(*t*) depends only weakly on such parameters as *M*, *R*, and *T* but is strongly dependent on Γ_n_. Thus, to enhance the density gain and signal visibility in cell molasses it is important to select an atom with a large transition width. Alternately, it may be possible to achieve this artificially, possibly by power broadening the transition, if this can be done without greatly degrading the cooling process.

The above analysis has assumed a two-state atom. In actuality since Na has two ground states (*F* = 1 and *F* = 2), two frequencies are necessary to prevent optically pumping the atom to the other ground state and losing it to the cooling process. The two-state ideal can be approximated by having both frequencies present in the molasses region. This can be accomplished by two different arrangements. The first uses both frequencies in all beams. This is the usual arrangement for optical molasses in beam experiments. The second arrangement uses one frequency in two pairs of beams with the other frequency in the third pair of beams. This second optical arrangement, by separating the frequencies, nearly eliminates the effect of the surrounding 1-D molasses region while possibly enhancing the signal-to-background fluorescence ratio [7]. One particular arrangement might be to have only light resonant with the *F* = 2 to *F*′ = 2 transition in two pairs of counter propagating beams, while the third pair contains only light resonant with the *F* = l to *F*′ = 2 transition. With this setup, the cooling in the 1-D molasses regions would be effectively turned off after only a few transitions as the atom is optically pumped to the other ground state. Only in the intersection of the beams would both frequencies be present to prevent optical pumping out of the cooling process. The triangular points of figure 2 show the result of optical pumping, the lower velocities become catchable again as the effect of the 1-D molasses region is nearly eliminated. For this arrangement, the cooling in the intersection region will proceed at a reduced rate (since cooling on the *F* = 2 to *F′* = 2 transition is not as effective for not clearly understood reasons), but 3-D cooling will still occur.

The big advantage with this technique is that the visibility should be greatly enhanced. While both the cold atom signal would be smaller (due to less effective damping using these transitions) and the background signal would be smaller (due to optical pumping), it is expected that the background signal reduction would be greater than the cold atom signal reduction. The rms thermal velocity of the atoms in the cell is about 6 × 10^4^ cm/s. This gives a transit time across the 1 cm beams of 1.6 × 10^−5^ s. Using either the *F* = 1 to *F*′ = 2 or *F*′ = 2 to *F*′ = 2 frequency alone will optically pump the atoms after only a few transitions or about 100 ns. Since a hot atom is in the beam for 160 times as long as this optical pumping time, the background fluorescence should be reduced by nearly this factor.

## 4. Experiment

To look for evidence of molasses experimentally, we set up a Na vapor cell with a base pressure at room temperature of 2 × 10^−10^ Torr. The cell was operated at 75 °C. An effort was made to make the temperature distribution as uniform as possible. This uniformity was important to prevent a non-Maxwell-Boltzmann velocity distribution resulting from local hot spots in the cell. The Na vapor pressure at this temperature was measured to be in the range of 1 to 3 × 10^−8^ Torr. The cell windows were antireflection coated to reflect less than 0.25% per surface. High reflectivity (*R*>99.85%) mirrors were used to retroreflect the beams. Each of the three mutually orthogonal pairs of counterpropagating beams were linearly polarized with their polarizations mutually orthogonal. The Na D_2_ line was used for the cooling transition. The power in each beam was varied from about 1 to 10 mW with the beams apertured from 0.5 to 1 cm in diameter. The collimation of the molasses beams were set using a shearing interferometer. With this interferometer, we were able to set the radius of curvature of the wavefront to be greater than 100 m. A photo-multiplier tube was used to collect the light from the region of intersection of the beams.

In setting up this experiment, there is the practical problem of finding the initial evidence of a signal in the presence of the background fluorescence. It is our experience that in a beam experiment, molasses lifetimes of about 100 ms can be set up blind (i.e., with no need to see the signal). For molasses in a cell, the maximum lifetime may be somewhat smaller than this due to collisions with background atoms, so we need a signal that is visible at this lifetime to observe the effect. Our calculations of density buildup are valid when the rate of increase is greatest, that is, for times much less than any of the limiting lifetimes. Therefore, we chose to look for density buildups on a time scale of less than about 50 ms. We attempted to observe the signal (with both frequencies in all beams) using a lock-in amplifier and modulating the *F* = 1 to *F* = 2 laser frequency on the order of 10 MHz at a 20 Hz rate while slowly scanning the *F* = 2 to *F* = 3 laser. This would allow the cold atom signal to build up for 25 ms. According to our calculations, using *V*_c_ = 20 m/s, a 1-cm region, and allowing for the reducton due to 1-D molasses surrounding the central 3-D region, the initial rate of density increase for the above parameters is about 3.5% per second. Since the background signal results from the fluorescence of about 3.6% of the total number of atoms (for our Na cell at 75 °C, the natural width is ~0.7% of the Doppler width), the rate of increase in cold atom fluorescence signal is predicted to be about 100% of the background signal per second. For the 25 ms build up time, the expected fluorescence increase due to laser cooled atoms is approximately 2.5% over the background fluorescence. The signature of this signal should be a peak with a frequency width on the order of the natural width of the transition and located just to the red of the *F* = 2 to *F* = 3 transition. The signal should, of course, disappear when one of the six beams is blocked. We estimate that our sensitivity is roughly at the 1*%* level, which is comparable to our expected signal. We saw no evidence of laser cooled atoms in the cell using the above setup, or the alternate arrangement of separate frequencies in separate beams, so we are only able to conclude that our signal estimates give a limit to the size of the cold atom density buildup.

## 5. Discussion

One important assumption in our calculations is that the velocity distribution is quickly rethermalized as the low velocity atoms are caught in the molasses. In our cell, the temperature and density are such that, for thermalization purposes, there are essentially no collisions between atoms in the gas phase. In addition, the sticking coefficient for Na atoms hitting the walls should be nearly unity. This means that any atom in the gas is a fresh atom emitted from the wall with no memory of any previous velocity. As a result, our calculations depend on the velocity distribution of the atoms emitted from the walls being Maxwell-Boltzmann. This should be a valid assumption since, at the low densities of our cell, the low-velocity end of the distribution should not be suppressed. This is in contrast to beam experiments, where the low velocity atoms can be depleted by collisions with a large flux of faster atoms [8]. We mention here, for completeness, that there may be other mechanisms that could drastically reduce the rate of emission of slow atoms from the walls, such as a surface barrier potential. If such a barrier did exist, the slowest atoms emitted by the wall would not make it over the hill to enter the molasses volume. In this case, rethermalization would occur through inelastic interactions between the atom and the barrier. This could be a very slow process which would reduce the cold atom density that could be achieved. Also, if for some reason the sticking coefficient was anomalously low, the rate of rethermalization would be slow, reducing the rate of cold atom buildup.

## 6. Conclusions

We have estimated the processes relevant to laser cooling atoms in a thermal vapor cell. We have shown which parameters are important in attempting to observe such cooling. This should be a useful guide when considering the relative merits of trying to cool other atoms or trying to cool on other transitions. Our experimental results give an upper limit on the size of the cold atom density buildup that can be expected for optical molasses in a thermal vapor cell. In particular, we have seen that it is most important to have a large width for the cooling transition. It is left open whether an appropriate system can be found with a broader transition or one that can be broadened artificially by some means, such as power broadening, which will improve the cold atom density increase and allow cell molasses to achieve its promise as a convenient source of cold atoms.
